# Retrospective Study of Midazolam Protocol for Prehospital Behavioral Emergencies

**DOI:** 10.5811/westjem.2020.3.45552

**Published:** 2020-04-21

**Authors:** Ryan M. Huebinger, Hashim Q. Zaidi, Katie L. Tataris, Joseph M. Weber, Kenneth S. Pearlman, Eddie Markul, Leslee Stein-Spencer, Christopher T. Richards

**Affiliations:** * McGovern Medical School at The University of Texas Health Science Center at Houston (UT Health), Department of Emergency Medicine, Houston, Texas; † University of Chicago Pritzker School of Medicine, Section of Emergency Medicine, Chicago, Illinois; ‡ Chicago Emergency Medical Services System, Chicago, Illinois; § John H. Stroger, Jr., Hospital of Cook County, Department of Emergency Medicine, Chicago, Illinois; ¶ Northwestern University Feinberg School of Medicine, Department of Emergency Medicine, Chicago, Illinois; || Advocate Illinois Masonic Medical Center, Department of Emergency Medicine, Chicago, Illinois; # Illinois Department of Public Health, Springfield, Illinois; ** Northwestern Feinberg School of Medicine Center for Healthcare Studies, Institute for Public Health and Medicine, Chicago, Illinois; †† University of Cincinnati School of Medicine, Division of EMS, Department of Emergency Medicine, Cincinnati, Ohio

## Abstract

**Introduction:**

Agitated patients in the prehospital setting pose challenges for both patient care and emergency medical services (EMS) provider safety. Midazolam is frequently used to control agitation in the emergency department setting; however, limited data exist in the prehospital setting. We describe our experience treating patients with midazolam for behavioral emergencies in a large urban EMS system. We hypothesized that using midazolam for acute agitation leads to improved clinical conditions without causing significant clinical deterioration.

**Methods:**

We performed a retrospective review of EMS patient care reports following implementation of a behavioral emergencies protocol in a large urban EMS system from February 2014–June 2016. For acute agitation, paramedics administered midazolam 1 milligram (mg) intravenous (IV), 5 mg intramuscular (IM), or 5 mg intranasal (IN). Results were analyzed using descriptive statistics, Levene’s test for assessing variance among study groups, and t-test to evaluate effectiveness based on route.

**Results:**

In total, midazolam was administered 294 times to 257 patients. Median age was 30 (interquartile range 24–42) years, and 66.5% were male. Doses administered were 1 mg (7.1%) and 5 mg (92.9%). Routes were IM (52.0%), IN (40.8%), and IV (7.1%). A second dose was administered to 37 patients. In the majority of administrations, midazolam improved the patient’s condition (73.5%) with infrequent adverse events (3.4%). There was no significant difference between the effectiveness of IM and IN midazolam (71.0% vs 75.4%; p = 0.24).

**Conclusion:**

A midazolam protocol for prehospital agitation was associated with reduced agitation and a low rate of adverse events.

## INTRODUCTION

Agitated patients pose challenges for both patient care and emergency medical services (EMS) provider safety, but there is no consensus regarding the optimal medication to manage prehospital agitation.^1^ Behavioral emergencies are complex with numerous etiologies, including neurologic, traumatic, intoxication, acute psychiatric, infectious, and metabolic.^2^ While EMS providers can use de-escalation techniques and physical restraints, pharmacologic intervention may be required when non-pharmacologic methods fail to effectively control agitation.^3^,^4^

Previous studies in the emergency department (ED) setting demonstrate that benzodiazepines and antipsychotics can be effective agents todecrease patient’s level of agitation.^5^–^7^ When given alone, benzodiazepines have a well-established safety profile, a rapid onset of action, and are effective in treating agitation.^8^,^9^ Specifically, midazolam provides a quicker onset when compared to lorazepam and haloperidol while maintaining equivalent sedative potency.^6^,^10^,^11^ Additionally, midazolam can be administered via the intranasal (IN) route as well as the intravenous (IV) and intramuscular (IM) routes.^12^ The IN route offers a needle-less option for EMS providers, thus decreasing the risk of blood-borne pathogen exposure.^13^,^14^ While IN midazolam has been proven to be safe and effective when administered to control seizures and for procedural sedation, there is limited research regarding its use for prehospital behavioral emergencies.^15^,^16^ Prior studies have described the IM and IV routes for chemical sedation, but IN administration remains under-studied.^4^,^7^

In this study, we report the rate of clinical improvement, need for repeat dosing, and rate of adverse events for a prehospital behavioral emergencies protocol using midazolam. Secondarily, we compare the effectiveness and safety of IN midazolam to IM midazolam for treating behavioral emergencies.

## METHODS

### Study Design, Population, and Setting

We performed a retrospective chart review of patients who were administered midazolam by EMS for behavioral emergencies from February 2014 through June 2016. We conducted this study in the Chicago EMS System, which serves an estimated 2.7 million residents and covers 237 square miles. The Chicago EMS System is a regional collaborative of hospital-based, EMS physicians and nurses who provide medical oversight for EMS provider agencies within the system, including the Chicago Fire Department (CFD), which provides emergency response to all 9-1-1 calls. CFD is an urban, fire-based EMS agency with over 280,000 annual transports, of which approximately 3% are for behavioral or psychiatric emergencies and related complaints.

In 2014, the Chicago EMS system implemented a new protocol for management of patients with behavioral emergencies using midazolam. Paramedics had previous training on the use of IN medications and on the use of IV midazolam for other indications. They underwent additional training for the use of midazolam for behavioral emergencies including IN delivery. Per protocol, paramedics attempted verbal de-escalation techniques and physical restraint, but if a patient remained combative and physically dangerous to themselves and others, paramedics could administer midazolam. Midazolam dosing was 1 milligram (mg) IV or 5 mg either IM or IN (repeating once as needed), guided by prior studies.^7^,^10^,^11^,^20^,^21^ EMS providers then documented the dose and route of midazolam administration in addition to the patient’s response to therapy with one of the following options: a) clinical deterioration; b) no change; c) slight improvement; or d) significant improvement. Paramedics also documented the indication for midazolam administration as either “behavioral emergency” or “seizures” in the electronic patient care report.

**Population Health Research Capsule**

What do we already know about this issue?

While prehospital agitation poses a significant challenge to emergency medical services providers, medications can help improve safety of care and transport.

What was the research question?

Is a prehospital protocol using midazolam for agitation safe and effective?

What was the major finding of the study?

Use of this protocol was associated with decreased agitation and had a low rate of complications.

How does this improve population health?

Most of the agitated patients in our study were from racial minorities. Identifying the optimal treatment for agitation is important for this at-risk population.

Using SafetyPAD software (ESO Solutions Inc, Austin, TX), we extracted all EMS patient care reports from February 2014 through June 2016 of patients for whom 9-1-1 was called and in which midazolam was administered. We included all cases in which midazolam was administered to adult patients for behavioral emergency via the IM, IN, or IV route. We excluded all cases in which midazolam was given for indications other than behavioral emergency, cases in which midazolam was administered other than via IM, IN, or IV routes, and cases with dosages outside the range prescribed in the protocol. Per EMS patient care protocol, we excluded patients less than 18 years of age or greater than 60 years of age. Additionally, we excluded cases if key data elements were missing, such as dose, route, or patient response.

Patient demographic information including age, gender, and race was collected and included in analysis. Additionally, an unblinded abstractor (RH) reviewed all charts for complications, predefined as systolic blood pressure < 100, oxygen saturation < 95%, use of airway intervention, and mention of provider injury. RH was trained in chart abstraction by another author (CTR). Meetings were held weekly during chart abstraction to answer questions and review results.

### Outcomes and Analytical Methods

We evaluated the dose, route, need for repeat dosing, and clinical effect of midazolam administrations, and we performed descriptive statistics for administrations of midazolam for behavioral emergencies. Levene’s test was used to assess variance between aggregate groups of “any improvement” (significant and slight improvement) and “no improvement.” Using t-tests, we compared effectiveness between the routes and rate of adverse events for IN and IM administrations. We used Stata 15 (StataCorp, College Station, TX) to create descriptive statistics, calculate confidence intervals (CI), and perform the t-tests.

### Human Subjects Committee Review

Northwestern University’s institutional review board approved the study.

## RESULTS

**Figure 1:**
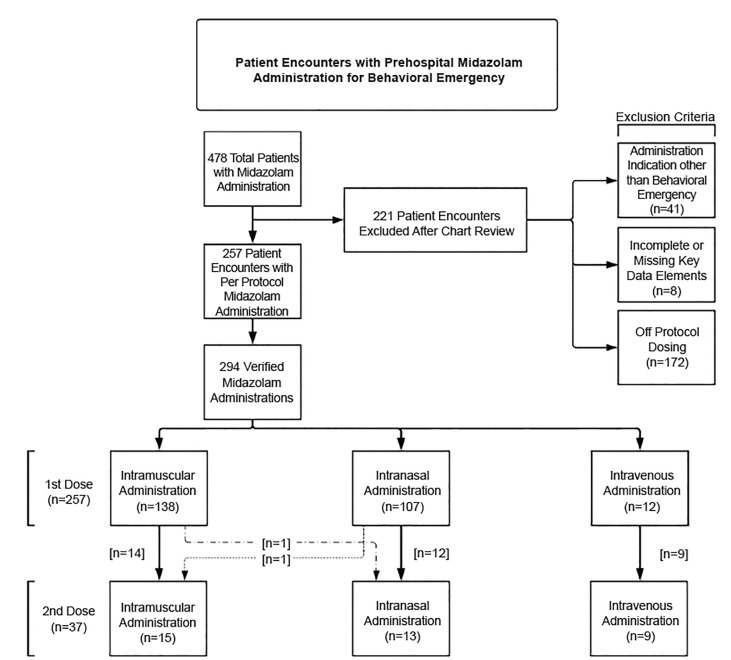
Patient encounters reviewed for midazolam administration in prehospital.

During the study period, 478 patients received midazolam. We excluded 221 cases for indications other than behavioral emergency, deviations from dosing protocol, or missing data (Figure 1). After exclusions, we included 294 administrations to 257 patients. Patient characteristics are reported in Table 1.

Including all administrations of midazolam (n = 294), paramedics noted improvement in 73.5% of cases, 34.5% (95% CI, 29.6–39.8%) of which had substantial improvement in level of agitation and 39.3% (95% CI, 34.2–44.7%) had slight improvement. No improvement was noted in 25.5% (95% CI, 21.1–30.5%) of cases, and 0.6% (95% CI, 0.1–2.4%) had clinical deterioration. Of all administrations, 52.0% were IM, 40.8% were IN, and 7.1% were IV. The doses administered were 1 mg (7.1%) and 5 mg (92.9%).

**Figure 2:**
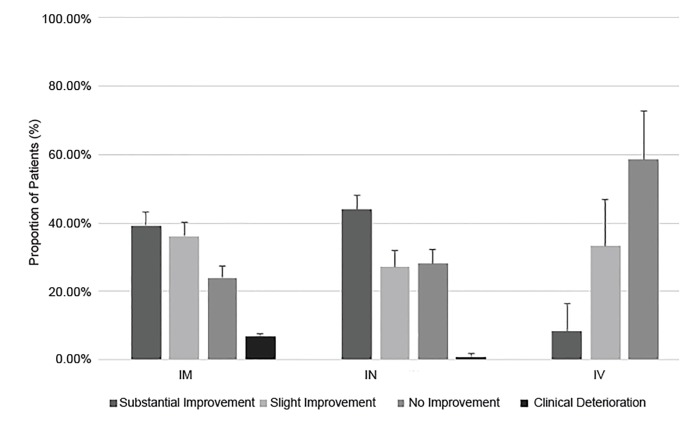
Subjective clinical change after initial midazolam administration for behavioral emergencies as reported by paramedics. *IM*, intramuscular; *IN*, intranasal; *IV*, intravenous.

In the subset of first-dose midazolam administrations (n = 257), paramedics reported substantial improvement in 32.7% (95% CI, 27.2–38.7%), slight improvement in 39.3% (95% CI, 33.5–45.4%), no change in 27.2% (95% CI, 22.1–33.0%), and deterioration in 0.8% (95% CI, 0.1–3.1%) (Figure 2). The routes were IM (53.7%), IN (41.6%), and IV (4.7%). The majority of first doses were 5 mg. (Table 2). Response rates to specific routes are shown in Figure 2.

**Figure 3:**
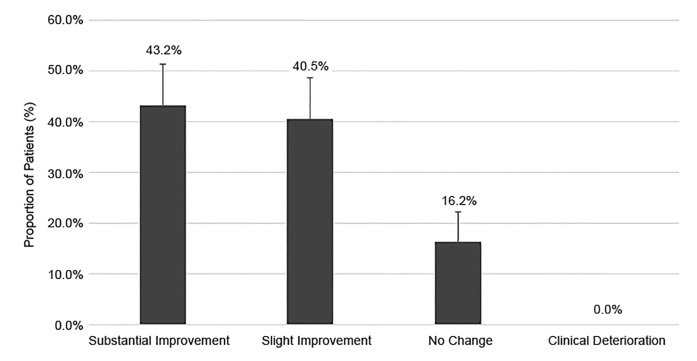
Subjective clinical change after repeat midazolam administration for behavioral emergencies as reported by paramedics.

In those patients requiring a repeat dosage (n = 37), paramedics reported improvement in 83.7% of cases, with substantial improvement noted in 43.2% (95% CI, 28.0–59.9%) of patients, and slight improvement documented for 40.5% (95% CI, 25.7.0–57.3%) of patients. No change was noted in 16.2% (95% CI, 7.3–32.4%) of patients (Figure 3). The routes of the second dose were IV (24.3%), IM (40.5%), and IN (35.1%) (Table 2). Of those receiving a second dose, their responses to the first dose were as follows: no response (75.7%, 95% CI, 58.9–87.1%); slight improvement (18.9%, 95% CI, 9.1–35.3%); substantial improvement (2.7%, 95% CI, 0.4–17.9%); and clinical deterioration (2.7%, 95% CI, .4–17.9%).

IM and IN routes of midazolam administration were compared for effectiveness after applying Levene’s test to compare “any improvement” (“slight improvement” + “substantial improvement”) to “no improvement” (“no improvement” + “clinical deterioration”). The datasets were found to be homogenous (p = 0.18). Using a t-test, we found no significant difference between “any improvement” after IN midazolam (71.0%) and IM (75.4%, p = 0.24). There was also no significant difference found between the documented adverse events of IM midazolam (3.9%) and IN (3.3%, p = .79). Additionally, we found no significant difference between the rates of reported EMS provider injury for IM (3.9%) and IN (1.7%, p=.27) doses. The majority of these injuries were kicks or bites by the patient, with no needlestick injuries reported.

Paramedics reported six adverse events thought to be due to midazolam administration, and an additional three adverse events were identified upon chart review. Adverse events included hypotension (systolic blood pressure < 100 millimeters of mercury [mmHg]) (n = 3); hypoxia with airway intervention required (n = 1); hypoxia without airway intervention (n = 1); unresponsiveness (n = 2); traumatic cardiac arrest (n =1); and worsening agitation (n = 2). For the nine cases, all happened after a single 5 mg dose administered via IM (n = 6) or IN (n = 3) routes. The patient who received an airway intervention had an oropharyngeal airway placed and bag-valve-mask ventilation performed. In the two cases of hypotension with systolic pressures less than 100 mmHg, none of the cases had a systolic blood pressure less than 90 mmHg systolic. Of patients experiencing unresponsiveness, two were given midazolam for agitation after naloxone was administered for suspected opioid overdose. The patient who experienced traumatic cardiac arrest sustained blunt trauma injuries after a four-story fall. This patient received 5 mg of midazolam IM to facilitate safe and timely transport in the setting of severe trauma and experienced cardiac arrest during transport.

## DISCUSSION

To our knowledge, this study represents the largest cohort of prehospital patients administered midazolam as a single agent for behavioral emergency. Paramedics reported clinical improvement in a majority of patients following midazolam administration. Only 14.4% of patients required a second dose, after which, the majority were assessed by paramedics to have a clinical improvement. Over 294 administrations, adverse events were noted in 3.1%, all with IM or IN dosing. Based on this improvement rate and low complication rate, a prehospital behavioral emergencies protocols using midazolam to control agitation may be considered for use in EMS systems.

One notable advantage of midazolam over alternative agents is the possibility for IN administration. In this study, IN administration represented 40.8% of the midazolam administrations, suggesting a preference of IN route by EMS providers. Additionally, we found IN midazolam to be no less effective than IM midazolam. These results suggest that IN administration may represent a preferable route of delivering midazolam, particularly as the IN route eliminates the risk of needlestick provider injury.

Adverse events after midazolam use for behavioral emergency were rare in this study, with only 3.1% of patients experiencing an adverse event, all after initial administration of midazolam. Hypoxia and apnea were also rare, with only one patient requiring any airway intervention and no patients requiring intubation in the field. For the patient administered midazolam in order to facilitate transport in the setting of blunt trauma, they likely experienced cardiac arrest due to injuries rather than midazolam administration. Further studies are needed to investigate the safety of midazolam administration in the setting of known or suspected opioid use as prior studies have demonstrated that concurrent benzodiazepine and opioid administration increases the risk of respiratory depression.^17^–^19^

Prehospital providers are frequently exposed to agitated patients, and improved strategies are needed to safely care for these patients.^3^,^14^ The optimal agent for safely managing agitation in the prehospital setting after de-escalation techniques have failed remains to be determined. Benzodiazepines are one of the most frequently used classes of drugs for acute agitation due to their safety profile and sedating effects. IM doses of short-acting benzodiazepines like midazolam have shown rapid onset of action and more rapid effect when compared to antipsychotics alone.^6^,^7^,^10^,^20^ However, sedating effects from benzodiazepines have raised concerns about potential respiratory depression and their use may lead to an increase in respiratory adverse events.^6^,^7^,^20^,^21^

An alternate agent for prehospital agitation that has attracted attention over the last decade is ketamine. Despite being used since the 1960s, ketamine has only recently been evaluated for use in treating agitation in the prehospital and ED settings and has been shown to be effective in controlling agitated patients in several studies.^22^–^24^ However, research on the use of ketamine in the prehospital setting has demonstrated hypoxia, increased secretions, and laryngospasm requiring intubation following ketamine administration.^22^–^25^ In one of the largest studies to date evaluating ketamine for prehospital agitation, ketamine performed well in comparison to haloperidol in controlling agitation but with an intubation rate of 39%.^23^ While further studies are needed to clarify the use of ketamine for prehospital agitation, alternatives such as benzodiazepines may be preferable given the low frequency of complications requiring advanced airway as shown in this and other studies.^6^,^10^

## LIMITATIONS

Using paramedic impression as an outcome limits the results of this study. While paramedic impression is certainly important for prehospital treatments and has been used in prior studies, using a standardized aggression scoring systems may more accurately measure effectiveness of midazolam in treating agitation and improve external validity.^24^,^26^ A large portion of patients receiving 1 mg of IV midazolam required a second dose. This likely represents an under-dosing by the protocol, and the study EMS system has subsequently implemented a change to 2 mg for IV doses. We did not investigate the need for additional sedation in the ED, and we look forward to future studies linking prehospital and ED data.

We did not limit midazolam administration to excited delirium, so these results may not accurately represent the effects of midazolam on excited delirium. Excited delirium represents a small portion of agitated patients though, and protocols are necessary for control of agitation in a variety of clinical scenarios. Additionally, despite 3% of transports being for behavioral complaints, less than 0.1% of patients received midazolam during the study period. This likely suggests that non-pharmacological approaches may be adequate to address the majority of behavioral emergencies.

Adverse events were limited to paramedic documentation and chart review, which may not capture all adverse events, particularly paramedic injuries. We excluded more than a third of cases for dosing deviations, the large portion of which were due to online medical control. Excluding these deviations might bias the results by missing adverse events, but on analysis of all midazolam administrations, the adverse rate was similar at 2.9% for all administrations compared to 3.1% for per protocol. Lastly, this study was performed in a single EMS system, and further studies could verify effectiveness in other settings.

## CONCLUSION

In a large urban EMS system, we found that a prehospital behavioral emergencies protocol using midazolam was associated with improved agitation and a limited number of adverse events. Additionally, we demonstrate the effective use of IN midazolam for use in a prehospital behavioral emergencies protocol. Further studies will be needed to validate these findings in other EMS systems and to compare midazolam to other pharmacological options to help determine the ideal agent for the agitated prehospital patient.

## Figures and Tables

**Table 1:** Characteristics of patients receiving midazolam for behavioral emergencies.

Characteristic	Total patients (n = 257)
Age – median years (IQR)	30 (24–42)
Male gender	171 (66.5%)
Race	
African American	138 (53.7%)
White	65 (25.3%)
Hispanic	43 (16.7%)
Asian	6 (2.3%)
Other	2 (0.8%)
Unknown	3 (1.2%)

*IQR*, interquartile range.

**Table 2:** Characteristics of midazolam administrations for behavioral emergencies.

Administration Characteristic	Initial Dose (n = 257)	Repeat Dose (n = 37)
Dose administerd, n (%)		
1mg	12 (4.7%)	9 (24.3%)
5 mg	245 (95.3%)	28 (75.7%)
Administration route, n (%)		
IM	138 (53.7%)	15 (40.5%)
IN	107 (41.6%)	13 (35.1%)
IV	12 (4.7%)	9 (24.3%)

*IM*, intramuscular; *IN*, intranasal; *IV*, intravenous, *mg*, milligrams.
